# Mandated lowering of toxicants in cigarette smoke: a description of the World Health Organization TobReg proposal

**DOI:** 10.1136/tc.2007.024158

**Published:** 2008-03-28

**Authors:** D M Burns, E Dybing, N Gray, S Hecht, C Anderson, T Sanner, R O’Connor, M Djordjevic, C Dresler, P Hainaut, M Jarvis, A Opperhuizen, K Straif

**Affiliations:** 1 UCSD School of Medicine, San Diego, California, USA; 2 Division of Environmental Medicine, Norwegian Institute of Public Health, Oslo, Norway; 3 Cancer Council of Victoria, Melbourne, Victoria, Australia; 4 University of Minnesota Cancer Center, Minneapolis, Minnesota, USA; 5 Department of Environmental and Occupational Cancer, Institute of Cancer Research, The Norwegian Radium Hospital, Oslo, Norway; 6 Roswell Park Research Institute, Buffalo, New York, USA; 7 Tobacco Control Research Branch, National Cancer Institute, Bethesda, Maryland, USA; 8 Arkansas Department of Health, Little Rock, Arkansas, USA; 9 International Agency for Research on Cancer, Lyon, France; 10 Royal Free and University College London Medical School, London, UK; 11 National Institute of Public Health and the Environment (RIVM), The Netherlands

Preventing initiation of tobacco product use, promoting cessation of tobacco use, and protecting the public from exposure to second hand smoke are recognised by the World Health organization (WHO) Framework Convention on Tobacco Control (FCTC) and by the WHO Study Group on Tobacco Product Regulation (TobReg) as the most effective approaches to reducing tobacco related morbidity and mortality. However, the FCTC also recognises the need for tobacco product regulation in articles 9 and 10 of the treaty. In order to inform that process TobReg has developed a series of reports that begin to provide a scientific foundation for tobacco product regulation.^1^^–^6 This paper summarises a proposal, and the considerations that led to it, developed by a joint International Agency for Research on Cancer (IARC) and WHO working group, and approved by TobReg, which presents performance standards for cigarettes and a strategy to use them to mandate a reduction in the toxicant yields for cigarette smoke.

## WHY A NEW APPROACH WAS NECESSARY AND OTHER APPROACHES CONSIDERED

The most common measurements used historically to categorise cigarette smoke have been machine measured tar, nicotine and carbon monoxide (TNCO) yields per cigarette based on the US Federal Trade Commission (FTC)/International Standards Organization (ISO) testing regimen. There is a current scientific consensus that these per cigarette yields do not provide valid estimates of human exposure or of relative human exposure when smoking different brands of cigarettes.^1^ ^7^^–^9 Communication of these measures to smokers as estimates of their exposure or risk creates harm by misleading smokers to believe that differences in exposures and risk are likely to occur with switching to cigarette brands with different machine-measured yields. This ongoing harm precludes continued acceptance of current regulatory strategies based on per cigarette machine measured TNCO levels and necessitates development of new regulatory approaches.

Machine smoking regimens other than the FTC/ISO regimen have also been examined, particularly ones with more intense puffing parameters and which block some or all of the ventilation holes in cigarette filters. Examples include those developed by the US State of Massachusetts and the Canadian Government. These regimens generally produce higher yields per cigarette and reduce the differences between brands in the yields. Nevertheless, these regimens continue to maintain a ranking of brands by tar and nicotine yield per cigarette, and the rankings by yield per cigarette using these more intense regimens also do not provide valid estimates of human exposure, or of the relative exposure, experienced by smokers when they smoke different brands of cigarettes. A single machine testing regimen produces a single set of toxicant yields. In contrast to the machine, individual smokers vary the puffing pattern with which they smoke different cigarettes of the same brand, and cigarette design changes can lead smokers to systematically change how they puff cigarettes. Thus, even yields using these more intense smoking regimens have the potential to mislead smokers when expressed per cigarette and the machine measured yields should not be used to support claims of reduced exposure or risk.

These limitations of machine measurements led to efforts to quantify actual human exposures in smokers by measuring biomarkers in blood, urine, and saliva. However, since these biomarkers are measured in human smokers they are influenced by characteristics of the individual, characteristics of the individual’s smoking behaviour, as well as by characteristics of the product smoked.^2^ ^8^ Distinguishing the differences in biomarker levels due to variations between products from the differences due to smoker behaviour (eg who uses the product and how they use it), is a formidable scientific challenge. Research is needed to resolve these issues in order to allow exposure biomarkers to become an effective tool for product regulation. The multiplicity of brands on the market, self selection of smokers who use different products, and differences in how smokers of different products use them, make the use of biomarkers of exposure in a regulatory strategy to monitor cigarette product differences problematic given the current level of scientific knowledge.

Characterisation of the differences in harm caused by different cigarettes would offer a powerful metric for product regulation. Markers of biologically effective dose (levels of toxicants in critical target organs or tissues) are likely to be developed and validated in the future, and they are expected to offer more precise measures of smoke uptake and better predictions of smoke toxicity.^7^ Measures of injury or biomarkers of disease risk are also likely to be validated in the future. They will allow more rapid assessment of differences in disease risks than is currently available from epidemiological approaches measuring disease outcomes. These advances may allow assessment of differences in risk between tobacco products in the future. Nevertheless, at this moment, none of these measures have been validated as reliable independent predictors of differences in tobacco related disease risk among smokers using different products.^10^

The limitations of measures of human exposure and human injury suggest that, for the near future, product regulatory approaches may be limited to measures of the differences between products in design characteristics, contents and emissions rather than measures derived from their human use.

It is generally assumed that product design characteristics, constituents and additives contribute to the toxicity of cigarettes, the addictiveness of the product and the likelihood that new smokers will start or confirmed smokers will quit. TobReg has examined the evidence supporting these assumptions and concluded that further research is likely to provide compelling evidence to establish these assumptions as true.^4^ ^5^ Nevertheless, the existing science base is currently not sufficient to allow regulation of these characteristics based on their effects on toxicity of the product either by establishing product performance standards or by prohibiting the use of specific design features or constituents.^4^ ^5^ In addition, TobReg felt that regulation of the end product of tobacco combustion (emissions) might be a more robust tool for assessing product performance than changes in individual design features or components, particularly given the limited understanding of the interaction cigarette design features and components have in influencing smoke toxicity.

These reviews led TobReg to the conclusion that chemical measurements of the smoke produced by machines, and their use as inputs for product hazard assessment, with all of their limitations, may be the limit of current scientific assessment of differences between brands that can be used for regulatory assessment of product toxicity. This conclusion is limited to product toxicity regulation and is not intended to preclude regulation or prohibition of products with specific characteristic that have other important public health effects, for example candy flavoured cigarettes or reduced ignition propensity cigarettes.

## THE PROPOSED APPROACH FOR MANDATED LOWERING OF TOXICANT LEVELS

TobReg recommends a strategy for regulation based on product performance measures with the goal of reducing toxicant levels in mainstream cigarette smoke measured under standardised conditions.^5^ ^6^ It recommends establishing levels for selected toxicants per mg nicotine and prohibiting the sale or import of cigarette brands that have yields above these levels. The purpose of normalising toxicant levels per mg nicotine is to shift the interpretation of the measurement away from the quantity of the smoke generated per cigarette, and away from the misleading use of TNCO values as measures of human exposure and risk. It moves towards product characterisation of smoke toxicity generated under standardised conditions. The toxicants currently recommended for mandated reductions by TobReg and proposed levels are presented in table 1.

**Table 1:** Toxicants recommended for mandated lowering

Toxicant	Level in μg/mg nicotine (international brands)*	Level in μg/mg nicotine (Canadian brands)†	Criteria for selecting the value
NNK	0.072	0.047	Median value of the data set
NNN	0.114	0.027	Median value of the data set
Acetaldehyde	860	670	125% of the median value of the data set
Acrolein	83	97	125% of the median value of the data set
Benzene	48	50	125% of the median value of the data set
Benzo[a]pyrene	0.011	0.011	125% of the median value of the data set
1,3-Butadiene	67	53	125% of the median value of the data set
Carbon monoxide	18 400	15 400	125% of the median value of the data set
Formaldehyde	47	97	125% of the median value of the data set

*Based on data from Counts *et al.*^11^

†Based on the data reported to Health Canada minus the brands with NNN/mg nicotine levels over 0.1, which eliminates most US and Gauloise brands (http://www.hc-sc.gc.ca/hl-vs/tobac-tabac/legislation/reg/indust/constitu_e.html).^12^

NNK, 4-(methylnitrosamino)-1-(3-pyridyl)-1-butanone; NNN, *N′*-nitrosonornicotine.

Available data on the variation in the toxicant levels for cigarette brands provided an initial set of observations used to identify levels of reduction that have already been achieved by some products on the existing market. The initial levels suggested for regulating tobacco specific nitrosamines (NNK (4-(methylnitrosamino)-1-(3-pyridyl)-1-butanone) and NNN (*N′*-nitrosonornicotine)) are the median values for the brands on the market, and for seven additional toxicants the levels recommended are set at 125% of the median value of the toxicant per mg nicotine for the brands on the market being regulated. These initial levels are intended to be a first step in an overall strategy to further reduce levels of toxicants in tobacco smoke as our understanding of what is possible expands and new technology develops. The levels recommended by TobReg reflect a judgment from available data as to the most practical trade-off considering the need to regulate a range of toxicants, to mandate substantive lowering of those toxicants, and yet not to require elimination of most of the brands. Regulators are encouraged to obtain data from their own markets and of course may select different levels more appropriate for their own circumstances. The specific quantitative values recommended for the nine toxicants are derived from an international sample of Philip Morris (American blended cigarettes) and Canadian Brands (predominantly flue cured brands) to allow selection of values for the type of cigarette used in a market, and to help guide regulators who choose not to examine the variation in their own markets.

The recommendation is to implement the policy change in phases beginning with a period of required annual reporting of toxicant levels by cigarette manufacturers to the regulatory authority. This should be followed by the promulgation of the levels for toxicants above which brands cannot be offered for sale. Finally, the established levels would be enforced. It is expected that regulators will take additional actions to further reduce the mandated levels of toxicants as this regulatory strategy is fully implemented. These actions can be in the form of setting targets or milestones based on what may be achievable with new technology or product designs.

Prohibiting consumer communications based on *any* machine measurements is a necessary condition of this strategy. Given the limitations of existing science, regulatory authorities have an obligation to ensure that the public is not misled by the results of the recommended machine testing and mandated lowering regulatory strategy, as the public was misled by the use of machine testing for tar and nicotine yields.

## SELECTION OF THE MACHINE SMOKING REGIMEN

**Figure 1:**
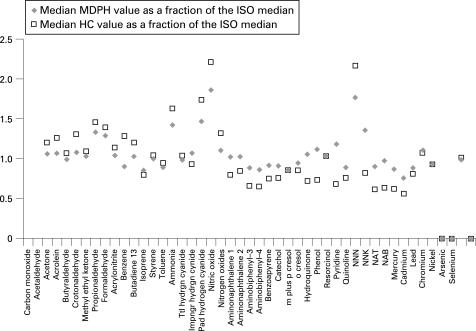
Ratio of the toxicant yield per mg nicotine for measurements using the Health Canada (HC) intense method and the Massachusetts Department of Public Health (MDPH) method compared to the International Standards Organization (ISO) method. ♦ Median MDPH value as a fraction of the ISO median, □ median HC value as a fraction of the ISO median.

Normalisation of the machine generated yields per mg nicotine, or per mg tar, does not eliminate the variation in the values measured by the different machine regimens. Figure 1 presents the values for toxicants in a data set of international Philip Morris brands measured with the standard ISO, the intense modified ISO used by Health Canada, and the Massachusetts Department of Public Health regimens. The data are presented as the ratio of the latter two regimens to the values derived using the standard ISO/FTC regimen. The differences in the yields of these toxicants per mg nicotine with these different regimens likely reflect differences in temperature of combustion, rates of air flow at the point of combustion and other factors that result from the differences in puff profiles used.

While these data demonstrate that full characterisation of the composition of the smoke generated by different brands of cigarettes would likely require a wide range of puff profiles, a performance standard is not intended to be a complete characterisation of the smoke but rather a standardised measure to allow regulatory comparison across brands in their performance under standardised conditions.

Performance standards could be set based on any machine regimen, but TobReg felt that recommending one regimen would reduce regulatory costs and promote international comparisons. All machine measurement regimens have limitations and no one regimen is ideal; but in considering the three regimens for which there are substantial regulatory and laboratory experience, the intense smoking regimen used by Health Canada was selected as having the best balance of advantages and limitations. This selection was based on several criteria. First, the larger quantity of smoke generated by this regimen reduces the coefficient of variation (CV) of the replicate measurements for some of the toxicants measured. Second, with certain design features the more intense machine parameters may yield levels of individual smoke toxicants substantially above those that would result with the ISO regimen. Third, the toxicants are generated under conditions of combustion that correspond to a more intense human smoking profile and therefore may better reflect how the product performs under these intense conditions. And fourth, the Canadian intense regimen may more accurately characterise cigarette design characteristics, such as charcoal filtration, where marked variation in yields result when the Canadian intense and standard ISO values are compared.

## SELECTION OF TOXICANTS

Data on comprehensive lists of toxicants measured in a consistent manner using the modified intense smoking regimen were available from three sources: a publication by Counts *et al*,^11^ which compares a set of international brands manufactured by Philip Morris, a set of Canadian brands^12^ reported by law to Health Canada for the year 2004, and a set of Australian brands^13^ provided to the Australian Department of Health and Ageing in the year 2001. The toxicants considered were confined to the list of toxicants reported in these data sets, but they represent most of the toxicants thought to play a major role in smoke toxicity.

The list of toxicants was prioritised by considering the known animal and human toxicity data for the compounds, toxicity indices based on the concentration of the constituent times its toxic potency factor, the variability of the toxicant across brands, the potential for the toxicant to be lowered in cigarette smoke with existing methods, and the need to have constituents that represent the different phases of smoke (gas and particulate), different chemical classes, and toxicities that reflect heart and lung disease as well as cancer. The most important criterion for selecting compounds for regulation is the direct toxicity evidence, but the toxicants were also compared by examining their hazard indices, which are generated by multiplying the yields per mg nicotine of individual smoke toxicants using the standard ISO machine smoking method with animal carcinogenicity and non-cancer potency factors, as a modification of the approach presented by Fowles and Dybing.^14^ Table 2 is an example of these indices calculated for the International set of Philip Morris brands.^11^

**Table 2:** Toxicant animal carcinogenicity and non-cancer response indices* per mg nicotine of constituents in smoke generated by the modified intense regime based on Counts *et al*^11^ 2005 smoke constituent level data

Constituent	Mean level in smoke, μg/mg nicotine	90th percentile in smoke, μg/mg nicotine	Max level in smoke, μg/mg nicotine	T25†mg/kg bw/day	Potency/mg/kg bw/day	Toxicant animal carcinogenicity index‡			Tolerable level μg/m^3^	Toxicant non-cancer response index§		
						Mean	90th percentile	Maximum		Mean	90th percentile	Maximum
Acetaldehyde	695	859	997	82.4	0.01	7.0	8.6	10.0	9	77.2	95.4	111
Acetone	359	446	501	ND	–	–	–	–	No TL	–	–	–
Acrolein	67.6	85.3	99.5	I	–	–	–	–	0.06	1127	1422	1658
Acrylonitrile	12.3	16.1	19.5	6.9	0.14	1.7	2.3	2.7	5	2.5	3.2	3.9
1-Aminonaphthalene	16.2¶	19.0¶	24.8¶	29.7	0.03	0.49×10^–3^	0.57×10^–3^	0.74×10^–3^	No TL	–	–	–
2-Aminonaphthalene	10.1¶	11.6¶	14.3¶	12.8	0.08	0.81×10^–3^	0.93×10^–3^	1.1×10^–3^	No TL	–	–	–
3-Aminobiphenyl	2.9¶	3.4¶	4.1¶	ND	–	–	–	–	No TL	–	–	–
4-Aminobiphenyl	2.2¶	2.7¶	3.2¶	ND**	–	–	–	–	No TL	–	–	–
Ammonia	21.2	26.8	40.7	ND	–	–	–	–	200	0.11	0.13	0.20
Arsenic	4.8¶	6.0¶	6.5¶	NQ	–	–	–	–	0.03	0.16	0.20	0.22
Benzene	39.0	45.8	51.1	13.4	0.07	2.7	3.2	3.6	60	0.66	0.76	0.85
Benzo[a]pyrene	9.0¶	11.2¶	13.8¶	1.1††	0.91	8.2×10^–3^	10.2×10^–3^	12.6×10^–3^	No TL	–	–	–
1,3-Butadiene	54.1	65.5	75.5	4.8	0.21	11.4	13.8	15.9	20	2.7	3.3	3.8
Butyraldehyde	43.0	52.4	63.6	ND	–	–	–	–	No TL	–	–	–
Cadmium	48.2¶	64.9¶	87.5¶	0.03	33	1.6	2.1	2.2	0.02	2.4	3.2	4.4
Carbon monoxide	15.2‡‡	17.9‡‡	27.3‡‡	ND	–	–	–	–	10 000	1.5	1.8	2.7
Catechol	48.8	61.6	65.4	104	0.01	0.49	0.62	0.65	No TL	–	–	–
Chromium	NQ	NQ	NQ	NQ	–	–	–	–	0.2§§	–	–	–
m-/p-Cresol	7.8	10.9	13.7	ND	–	–	–	–	600¶¶	0.01	0.02	0.02
o-Cresol	3.0	4.3	5.0	ND	–	–	–	–	600¶¶	0.01	0.01	0.01
Crotonaldehyde	28.8	36.6	41.3	I	–	–	–	–	No TL	–	–	–
Formaldehyde	41.1	57.9	90.5	NQ***	–	–	–	–	3	13.7	19.3	30.2
Hydrogen cyanide	204	277	390	ND	–	–	–	–	9	22.7	30.8	43.3
Hydroquinone	56.6	76.1	85.0	61.0	0.02	1.1	1.5	1.7	No TL	–	–	–
Isoprene	459	551	746	94.8††	0.01	4.6	5.5	7.5	No TL	–	–	–
Lead	23.5¶	27.7¶	48.6¶	39.3†††	0.03	0.00	0.00	0.00	No TL	–	–	–
Mercury	3.4¶	4.7¶	5.5¶	L	–	–	–	–	0.09	0.04	0.05	0.06
Methyl ethyl ketone	93.2	116	124	ND	–	–	–	–	1000	0.09	0.12	0.12
NAB	13.4¶	21.0¶	23.9¶	L	–	–	–	–	No TL	–	–	–
NAT	99.7¶	148¶	183¶	I	–	–	–	–	No TL	–	–	–
Nickel	NQ	NQ	NQ	NQ	–	–	–	–	0.05	–	–	–
Nitric oxide	180	280	349	ND	–	–	–	–	No TL	–	–	–
Nitrogen oxides	199	313	390	ND	–	–	–	–	40‡‡‡	5.0	7.8	9.8
NNK	70.1¶	102¶	111¶	0.015§§§	67	4.7	6.8	7.4	No TL	–	–	–
NNN	110¶	175¶	189¶	0.2	5.0	0.55	0.88	0.95	No TL	–	–	–
Phenol	11.4	17.1	19.8	I	–	–	–	–	200	0.06	0.09	0.10
Propionaldehyde	60.3	74.0	88.4	ND	–	–	–	–	No TL	–	–	–
Pyridine	21.4	25.4	28.1	L	–	–	–	–	No TL	–	–	–
Quinoline	0.33	0.42	0.46	ND	–	–	–	–	No TL	–	–	–
Resorcinol	1.0	1.4	1.6	I	–	–	–	–	No TL	–	–	–
Selenium	NQ	NQ	NQ	ND	–	–	–	–	20	–	–	–
Styrene	13.6	16.5	18.5	L	–	–	–	–	900	0.02	0.02	0.02
Toluene	71.1	83.3	96.3	E	–	–	–	–	300	0.24	0.28	0.32

*Toxicant animal carcinogenicity and non-cancer indices. A cancer hazard index (CHI) is the numerical value of the amount of an individual component in cigarette smoke, normalised per mg nicotine and multiplied with its cancer potency factor (1/T25). A non-cancer hazard index (NCHI) is the numerical value of the amount of an individual component in cigarette smoke, normalised per mg nicotine and multiplied with its non-cancer potency factor (or divided by its TL).

†Values are from oral administration unless otherwise stated.

‡Animal carcinogenicity potency factors. The T25 cancer potency estimation method of Dybing *et al*^16^ was used. The T25 value is the chronic daily dose, which will give tumours in 25% of the animals above background at a specific tissue site. The T25 is determined by linear extrapolation from the lowest dose giving a statistically significant increase in tumours (Dybing *et al*).^16^ The pertinent data from the individual cancer bioassay studies underlying the calculation of the T25 values are presented in the Annex of the full report. For the present calculations, the T25 values were converted to cancer potency factors per unit mg (1/T25).

§Non-cancer potency factors. Chronic reference exposure levels (REL) published by the Office of Environmental Health Hazard Assessment, California Environmental Protection Agency in February 2005 were used (http://www.oehha.ca.gov/air/chronic_rels/AllChrels.html). A chronic REL is an airborne level of a chemical at or below which no adverse health effects are anticipated in individuals indefinitely exposed to that level. RELs are developed both from human and animal toxicological data and presented referring to the target system for each substance. A REL is for the present calculations viewed as the inverse of the respective constituent’s non-cancer potency factor. In the present report, the term “tolerable level (TL)” was used instead of “REL”.

¶ng/mg nicotine.

**No data for T25 calculation.

††Inhalation administration.

‡‡mg/mg nicotine.

§§Hexavalent chromium.

¶¶Cresol mixture.

***Highly non-linear dose–response.

†††Lead subacetate.

‡‡‡WHO guideline for nitrogen dioxide, not listed by Cal/EPA in 2005.

§§§Subcutaneous administration.

Bw, body weight; E, evidence of non-carcinogenicity; I, insufficient evidence of carcinogenicity; L, limited evidence of carcinogenicity; NAB, *N*'-nitrosoanabasine; NAT, *N*'- nitrosoanatabine; ND, no data; NQ, not quantifiable; NNK, 4-(methylnitrosamino)-1-(3-pyridyl)-1-butanone; NNN, *N′*-nitrosonornicotine.

There are obvious limitations to the methodology used here for ranking individual cigarette smoke toxicants. Each measured toxicant is treated individually, such that the possibility of chemical interactions—either enhancing or inhibiting the hazardous properties of the smoke—is not taken into account. Further, it is obvious that these calculations have only been possible for those toxicants where index values have been estimated, and not for the rest of the some 4000 individual constituents in cigarette smoke.^15^ Since many of the potency factors have been derived from animal experiments, the obvious limitations in extrapolating from animal models to the human situation also apply. These limitations preclude use of these indices as quantitative estimates of the likely harm or risk of exposure to these different toxicants or for comparison of the relative risk or harm of different cigarette brands. They do, however, provide one useful set of metrics that can be considered in selecting which toxicants to regulate.

In addition to those toxicants recommended for mandatory lowering, an additional set of toxicants were considered high priority for disclosure and monitoring of their levels by brand. They are: acrylonitrile, 4-aminobiphenhyl, 2-aminonaphthalene, cadmium, catechol, crotonaldehyde, hydrogen cyanide, hydroquinone, and nitrogen oxides.

**Figure 2:**
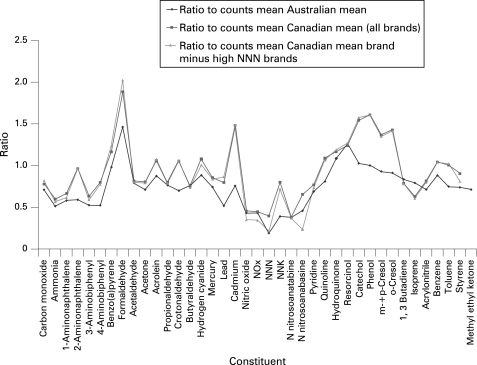
Ratio of mean for constituents for brands in different samples to the mean of the Counts *et al*^11^ sample.

Comparison of the mean levels of the specific toxicants across the different data sets for different countries helps define the differences in yield that result from the differences in the design of cigarettes offered for sale in different markets. Figure 2 presents the mean levels of each toxicant per mg nicotine, across all brands within the Canadian data set, the Canadian data set minus the US and French brands and for the Australian data. These means are presented as a ratio of the mean value for the toxicant per mg nicotine in the specified data set to the mean levels reported by Counts *et al*^11^ for the international Philip Morris brands. The magnitude of the variation in toxicant levels between the data sets is as large as, and sometimes exceeds, the variation in levels across brands within a data set. The magnitude of these variations suggests that examination of differences in toxicant yields for brands sold in different markets can offer an expanded understanding of the levels of individual toxicants potentially achievable with changes in cigarette design and manufacturing practices.

## THE BALANCE BETWEEN LEVELS, NUMBER OF TOXICANTS AND REMOVAL FROM THE MARKET

In the absence of a change in cigarette manufacturing processes, the number of brands potentially eliminated from the market by the proposed regulatory approach is dependent on the levels set and the number of toxicants regulated. Greater mandated reductions are possible where there is a high level of confidence that the levels of toxicants can be substantially reduced, as there is for tobacco specific nitrosamines, or when only a few toxicants are regulated. However, when only a few toxicants are regulated there is a greater probability that reductions in one toxicant might result in higher level of other toxicants in the brands remaining on the market as an unintended consequence. In considering this trade-off between numbers of toxicants regulated, levels of mandated reduction and numbers of brands eliminated from the market, TobReg felt that regulating a larger number of toxicants at the expense of accepting more modest levels of reductions reflected a more conservative initial step for regulation. As experience is accumulated with the capacity of the cigarette manufacturers to modify their products to achieve lower yields of specific toxicants, more aggressive targets for mandated lowering can be set by regulatory authorities.

## USES OF MANDATED LOWERING OF TOXICANT LEVELS IN A PRODUCT REGULATORY STRATEGY

The goal of the proposed regulatory strategy is to reduce the levels of toxic constituents measured under standardised conditions in the smoke of cigarettes allowed on the market. A secondary goal is to prevent the introduction into a market of cigarettes with higher levels of smoke toxicants than are present in brands already on the market.

**Figure 3:**
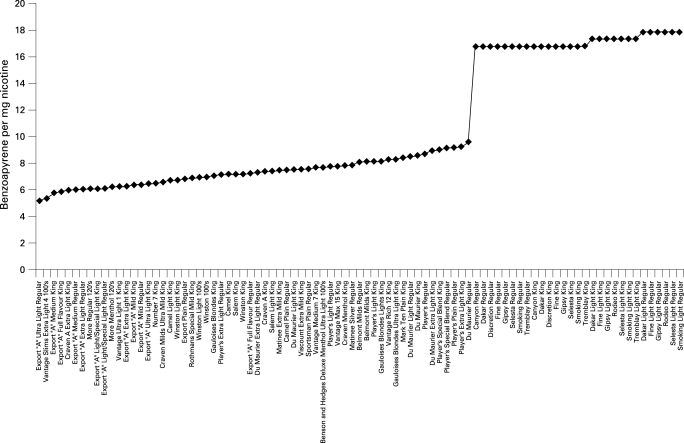
Benzoapyrene yield per mg nicotine in Canadian brands.

The value of examining the variation in toxicant yields for the market of interest rather than using an international sample is exemplified by comparing the benzo[a]pyrene yields in the international sample with those reported to Health Canada for Canadian brands (fig 3). When the international sample is ranked from the brand with the lowest level of benzo[a]pyrene/mg nicotine to the brand with the highest value, there is a continuous steady increase across the brands from 5.7 ng/mg nicotine to 13.8 ng/mg nicotine. By contrast, the ranking of Canadian brands from the lowest to the highest values presented in fig 3 reveals a level rising to approximately 9.6 ng/mg nicotine with a sudden jump to 16.8 ng/mg nicotine. The explanation for this sudden discontinuity in levels is not immediately evident, but the value of examining the Canadian experience rather than assuming that the sample of international brands would provide an adequate description of the Canadian market for establishing mandated reductions is evident.

**Figure 4:**
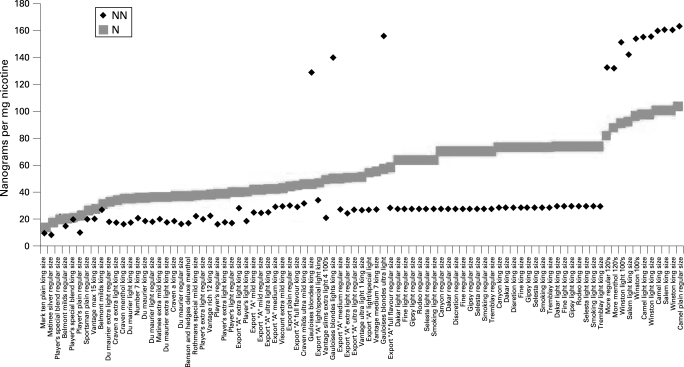
4-(methylnitrosamino)-1-(3-pyridyl)-1-butanone (NNK) and *N′*-nitrosonornicotine (NNN) by brand for Canadian cigarettes.

**Figure 5:**
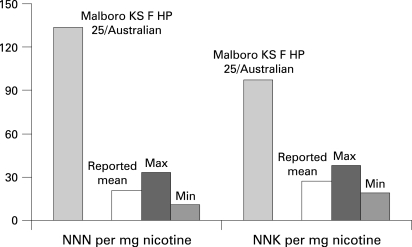
Mean and range of 4-(methylnitrosamino)-1-(3-pyridyl)-1-butanone (NNK) and *N′*-nitrosonornicotine (NNN) yields per mg nicotine for brands reported to the Australian government in 1999 contrasted with the levels of NNN and NNK reported for a Philip Morris Marlboro brand identified as being an Australian brand.

The possibility exists that, in the absence of effective product regulation, toxicant yields for the mix of brands on a market may actually increase as new brands are introduced or the characteristics of existing brands are changed. This possibility is of particular concern for those markets where current levels of toxicants are lower than those for brands sold in other markets. The data presented in fig 4 demonstrate that the brands reported to Health Canada with high NNK levels are largely brands also sold in the US and France, rather than those brands that are predominantly sold in the Canadian market. A similar circumstance exists in Australia and is presented in fig 5. The mean and range of NNN and NNK yields per mg nicotine for the brands sold in Australia (including Philip Morris brands) and reported to the Australian government in 2001^13^ are presented in the figure. They are contrasted with the much higher levels of NNN and NNK reported for a Philip Morris Marlboro brand identified as being an Australian brand in the paper by Counts and colleagues.^11^ To the extent that Marlboro becomes a leading brand in the Australian market, as it has in many other markets, the difference in NNN and NNK levels would be expected to increase the average yield for these toxicants of the cigarettes on the Australian market. Setting levels for toxicants would provide a regulatory strategy that could prevent the introduction of newer brands with greater toxicant yields than the existing brands.

## MONITORING FOR UNINTENDED INCREASES IN TOXICANT YIELDS

Mandating a reduction in some toxicants does not guarantee that levels of other, non-regulated toxicants will also be reduced, and it is possible that removal from the market of brands high in levels of the regulated toxicants will leave brands on the market with high levels of those toxicants not regulated. In addition, the changes in cigarette design and manufacturing implemented to lower the regulated toxicants may have the effect of increasing the levels of other non-regulated toxicants. TobReg recognised the potential for these unintended consequences of mandating reduction of specific toxicants and considered how monitoring might be put in place to detect them.

One approach would be to track the yields, or the percentage change in median yields, for the entire list of toxicants measured by Health Canada. However, different toxicants have different potency as toxicants, and the range of toxicant per mg nicotine (defining the amount of reduction possible with the proposed regulatory approach) is also different for the different toxicants. As a result, the net effect of a mandated reduction of some toxicants on total toxicant burden of the brands remaining will depend on the potency of the toxicant selected and the magnitude of the reduction achieved.

Based on these considerations, TobReg recommends using the sum of the individual toxicant animal carcinogenicity indices of the brands remaining on the market as a tool for examining the potential unintended consequences of regulation on net toxicant yields. TobReg chose this approach as a means of integrating animal toxicity data and toxicant level data of smoke generated under standardised conditions for the purpose of monitoring the effects of implementing the proposed regulatory approach on net toxicant yields. The sum of the toxicant animal carcinogenicity indices generated, and changes in that index, are mathematical constructs of toxicant yields and are based on animal toxicological evidence. They are not quantitative estimates of the human toxicity of the smoke of different brands or of the risk of developing cancer or other diseases in humans from smoking different brands, and they should not be applied in any quantitative way to estimating the risk of smoking or the overall toxicity of the smoke generated. They are recommended solely for use as a monitoring tool for regulators.

## CONSIDERATIONS FOR MODIFIED CIGARETTES AND POTENTIAL REDUCED EXPOSURE PRODUCTS

The recommendations in this report are intended to apply to traditional manufactured cigarettes that burn tobacco, and they should not be applied to cigarettes that heat tobacco or use technology other than combustion to deliver nicotine. Assessment of these unconventional tobacco products and other potential reduced exposure products (PREPs) are discussed in a previous WHO Scientific Advisory Committee on Tobacco Product Regulation (SACTob) report.^3^ It is possible to alter the level of a toxicant per mg nicotine in cigarette smoke by changing the nicotine yield of the cigarette, as well as by altering the level of the toxicant. The yield of nicotine in cigarette smoke can be increased by adding nicotine to the tobacco or the filter, as well as by using high-nicotine varieties of tobacco, among other approaches. While these approaches may theoretically have independent utility in decreasing exposure to tobacco toxicants, their potential to do so remains uncertain. Detection of increasing nicotine yields in brands can be facilitated by tracking of machine delivered nicotine yields per cigarette over time and by examining the distribution of tar to nicotine ratios for the brands within a given market. For those brands with increasing nicotine yields over time, and for those brands with tar to nicotine ratios in the bottom third of the brands on the market, regulators may choose to require that the brand be below a mandated limit value per mg tar as well as per mg nicotine. A similar adjustment to use per mg tar values can be made to those products that intentionally lower the nicotine content of the product.

## COMMUNICATION OF THE REGULATORY VALUES AND TESTING RESULTS TO THE PUBLIC

The mandated reduction of toxicant levels recommended in this report constitutes a first step toward improved tobacco product regulation. TobReg recognises the limitations of machine measurements and of setting levels based on per mg nicotine. Existing science does not allow a definitive conclusion that reduction of nitrosamines, or any other individual toxicants in cigarette smoke, will reduce cancer incidence, or the rate of any other tobacco related disease, in smokers who use cigarettes with lower levels of these toxicants, even though this is a hoped for outcome. Existing science has also not demonstrated that the specified changes in regulatory values will result overall in a meaningful change in actual exposure for consumers, although that is also a hoped for outcome. Mandating levels and disallowing brands with higher levels from the market is not a statement that the remaining brands are safe or less hazardous than the brands removed. It also does not represent governmental validation of the safety of the products that remain on the market. The proposed strategy for lowering toxicant yields is based on sound precautionary approaches of reducing toxicant levels where possible, similar to those used for other chemical consumer products.

Given the limitations of existing science, regulatory authorities have an obligation to ensure that the public is not misled by the results of the recommended machine testing and mandated lowering regulatory strategy, as the public was misled by the use of machine testing for tar and nicotine yields. TobReg notes that labelling of cigarettes with tar and nicotine and carbon monoxide levels measured by the ISO regimen persists and continues to be harmful to the public. TobReg recommends that any regulatory approach specifically prohibit the use of the results of the proposed testing in marketing or other communications with the consuming public including product labelling. It is also recommended that manufacturers be prohibited from making statements that a brand has met governmental regulatory standards, and from publicising the relative ranking of brands by testing levels. Because information is often transmitted to smokers through the kinds of news stories that accompany new regulation implementation, it is a responsibility of the regulatory structure to monitor the understanding of the consumer about the regulatory approaches undertaken. Regulators should pursue whatever corrective action is necessary to prevent consumers from being misled.

## SUMMARY

The WHO FCTC recognises the need for tobacco product regulation. It is incongruous that cigarettes, the single most hazardous consumer product, are not regulated as a product consistent with that hazard. Existing product regulatory strategies based on the machine measured tar, nicotine and carbon monoxide yields per cigarette are causing harm. Additional scientific research will be needed to develop validated measures of human exposure and risk that can be applied to individual brands; and, in the interim, performance standards based on machine measured emissions may be the limit of science-based regulation of individual brands. A strategy for regulation is proposed by the WHO based on product performance measures with the goal of reducing toxicant levels in mainstream cigarette smoke. It recommends establishing levels for selected smoke toxicants per mg nicotine and prohibiting the sale or import of cigarette brands that have yields above these levels. The toxicants selected were based on consideration of: animal and human toxicity data, hazard indices, variability of the toxicants across brands, the potential for the toxicant to be lowered, inclusion of constituents from both particulate and gas phases of smoke and from different chemical classes in cigarette smoke. Available data on the variation in the toxicant levels for cigarette brands are used to identify levels of reduction that have already been achieved by some products on the existing market. The recommended regulatory strategy should be implemented in phases beginning with a period of required annual reporting of followed by the promulgation of the regulatory levels for toxicants above which brands cannot be marketed and enforcement of those levels. Mandated lowering of levels of toxicants per mg nicotine in cigarette smoke will make regulation of cigarettes consistent with other regulatory approaches which mandate reduction of known toxicants in products used by humans. Use of the results of the testing, or of relative ranking of brands by testing levels, should be prohibited as are statements that the brand has met governmental regulatory standards.
